# Effects of Variable Dietary Sorghum Proportions on the In Vitro Digestibility of Nutrients for Ruminants

**DOI:** 10.3390/ani16050849

**Published:** 2026-03-08

**Authors:** Narimane Kellali, Iván Mateos, Cristina Saro, Nedjoua Lakhdara, Mustapha Kerrour, María José Ranilla

**Affiliations:** 1 Laboratoire Gestion de la Santé et Productions Animales, Institut des Sciences Vétérinaires, Université Frères Mentouri Constantine1, Constantine 25000, Algeria; lakhdara.nedjoua@umc.edu.dz (N.L.); kerrour.mustapha@umc.edu.dz (M.K.); 2 Departamento de Producción Animal, Universidad de León, E-24007 León, Spain; imata@unileon.es (I.M.); cristina.saro@unileon.es (C.S.); 3 Instituto de Ganadería de Montaña, CSIC-Universidad de León, Finca Marzanas s/n, E-24346 Grulleros, Spain

**Keywords:** sorghum forage, in vitro rumen fermentation, tannins, polyethylene glycol

## Abstract

This study evaluated the effects of increasing proportions of sudangrass sorghum forage in ruminant diets, with or without polyethylene glycol (PEG), on rumen fermentation, gas and methane production, nutrient digestibility, and protein fermentation metabolites. Three experimental diets containing 20%, 40%, or 60% sorghum forage (S20, S40, and S60) were incubated in vitro with cattle rumen fluid. Incubations were performed with or without PEG used as a tannin-binding agent. After 24 h of incubation, gas and methane production, in vitro dry matter digestibility (DMD), neutral detergent fiber digestibility (NDFD), ammonia nitrogen concentration (N-NH_3_), and volatile fatty acid (VFA) production and profiles were measured. Increasing sorghum inclusion resulted in a significant reduction in DMD (*p* = 0.0012). In contrast, NDFD increased (*p* = 0.0005), likely due to differences in lignin content among diets. Methane production was unaffected by the proportion of sorghum, despite the increasing tannin content. PEG supplementation significantly increased N-NH_3_ concentration (*p* = 0.042) and isobutyric molar proportion (*p* < 0.0001), indicating enhanced rumen protein degradation following tannin neutralization. The total VFA concentration was not influenced by either sorghum level or PEG treatment. However, higher sorghum inclusion was associated with shifts in the VFA profiles toward higher acetate (*p* = 0.0023) and lower butyrate proportions (*p* = 0.0114). Overall, the results suggest that moderate levels of condensed tannins (CTs) in sorghum forage may alter rumen fermentation patterns without markedly reducing methane production. PEG supplementation further confirmed the biological activity of tannins, especially regarding protein metabolism. Therefore, sudangrass sorghum may be considered a viable forage option for ruminant diets, provided its inclusion level and tannin effects are carefully managed.

## Simple Summary

In regions facing recurrent feed shortages, identifying locally available, drought-resistant forage resources is essential for sustainable ruminant production. Sorghum (*Sorghum bicolor* L. Moench) represents a promising alternative to conventional forages. However, its nutritional value may be limited by the presence of condensed tannins. Tannins are polyphenolic compounds with well-documented antinutritional effects, mainly due to their negative impact on feed digestibility in ruminants. This study investigated the effects of different dietary proportions of sorghum forage on in vitro rumen fermentation, as well as the role of polyethylene glycol as a tannin-binding agent. The results showed that higher sorghum inclusion altered rumen fermentation patterns, particularly volatile fatty acid profiles, and affected dry matter and fiber digestibility. In contrast, methane production remained unchanged. Polyethylene glycol supplementation further revealed the biological activity of tannins, particularly with regard to rumen protein degradation. Overall, sudangrass sorghum appears to be a viable forage option for ruminants in arid regions if its inclusion level and the effects of condensed tannins are appropriately managed.

## 1. Introduction

In North Africa, where fodder resources are largely derived from rangelands and cereal sub-products, livestock productivity is constrained by seasonal feed shortages and poor feed quality, especially during the dry season [1,2,3,4,5]. To mitigate these constraints, alternative feeding strategies have become essential [6], and extensive research has focused on identifying inexpensive, locally appropriate feed resources [7].

Among such potential alternatives, sorghum (*Sorghum bicolor* L. Moench) stands out as a promising option for ruminant feeding systems [8,9]. Sorghum is the fifth most important cereal crop globally, following wheat, rice, corn, and barley [10,11,12]. It is a drought-resistant cereal crop widely used for silage production to support dairy and meat output in ruminant systems [13]. As a forage crop, sorghum outperforms other cereals under various environmental stresses. It tolerates waterlogging, requires less water than corn and other common forage crops, and sustains higher yields in hotter climates, making it a more economical option [8,9,13,14].

In southern Algeria, sorghum has traditionally been cultivated for both human consumption (grains) and animal feeding (leaves and stalks) [15,16]. Expanding its cultivation into the north of the country, where livestock density is highest, could help offset the substantial fodder deficit, estimated at 5.2 billion fodder units [15].

However, sorghum is also characterized by a relatively high tannin content (10 g/kg DM) [17]. These compounds may limit its nutritional value due to their well-documented antinutritional effects [11,14,18]. Tannins are a heterogeneous group of polyphenolic secondary compounds widely distributed across the plant kingdom [19,20]. They are primarily known for their ability to bind and precipitate proteins, polysaccharides, vitamins, and minerals in environments with a moderate pH, such as the rumen [21,22,23,24,25]. Tannins have long been recognized as antinutritional compounds due to their negative impact on voluntary feed intake, ruminal fiber degradation, and overall feed digestibility in ruminants [24,26]. However, their effects can vary from beneficial to detrimental, depending on tannin type, chemical structure, molecular weight and the physiology of the target species [19,21,27]. Recent studies have highlighted the potential of tannins to positively modulate rumen fermentation. These effects include reduced ruminal protein degradation [28], increased availability of high-quality protein for absorption in the lower gut [29], and the prevention of bloat and reduced methane emissions [24,30].

Based on these considerations, evaluating the impact of varying proportions of sudangrass sorghum forage on rumen fermentation is essential to determine whether its tannin content significantly affects nutrient digestibility. Because tannins were not chemically quantified, the study focuses on evaluating their biological activity through PEG response rather than determining absolute tannin concentration. The present study therefore aimed to assess the in vitro effects of increasing dietary sorghum proportions, with or without polyethylene glycol (PEG), on gas production, methane emissions, nutrient digestibility, and protein fermentation metabolites in ruminant diets.

## 2. Materials and Methods

In this study, three experimental diets were formulated to contain 20%, 40%, and 60% fresh sudangrass sorghum forage (designated S20, S40, and S60). All diets were prepared using the same batch of ingredients: sorghum (S), alfalfa hay (L), corn silage (CS) and concentrate (C). Diets were standardized to maintain a constant forage-to-concentrate ratio of 60:40 across treatments. The concentrate was mixed in the laboratory and contained 55% wheat bran, 26% corn grain, 18% soybean meal, 1% mineral and vitamin supplements. The concentrate composition was kept identical across treatments to ensure that sorghum inclusion level was the only experimental variable. The composition of the three experimental diets is shown in Table 1.

Feed samples were oven-dried at 40 °C for approximately 48 h, ground through a 1 mm sieve, and subsequently analyzed for chemical composition. Organic matter (OM) content was determined according to the methods approved by the Association of Official Analytical Chemists (AOAC) [31]. Crude protein (CP) content was analyzed using the Kjeldahl method, as described in the AOAC [31]. The determination of neutral detergent fiber (NDF), acid detergent fiber (ADF), and acid detergent lignin (ADL) was performed following the method described by Van Soest et al. [32] using an ANKOM220 fiber analyzer unit (ANKOM Technology Corporation, Fairport, NY, USA).

In vitro digestibility was assessed using the batch culture technique described by Goering and Van Soest [33], with some adaptations [34,35] notably the use of ANKOM^®^ F57 filter bags (Macedon, NY, USA). Four different inocula were used for the in vitro incubations. Rumen fluid was collected at the slaughterhouse from four different cattle by manually squeezing the rumen contents. The rumen fluid was transferred to the laboratory in a preheated vacuum flask (39 °C) with an oxygen-free headspace and filtered through four layers of cheesecloth prior to use.

For each incubation, 500 mg of each preformulated diet was weighed into labeled ANKOM F57 filter bags, heat-sealed, and placed into 120 mL glass incubation bottles. Subsequently, 10 mL of rumen fluid diluted in 40 mL of Goering and Van Soest culture medium, prepared under continuous CO_2_ flushing, was added to each bottle. All bottles were hermetically sealed with rubber stoppers and aluminum crimps, and then incubated at 39 °C for 24 h. Each diet was incubated in quadruplicate.

Incubations were carried out in two separate runs (with or without the addition of 500 mg of PEG 6000) using the diverse inocula, yielding a total of eight replicates per sample. Sixteen additional bottles without substrate were included as blanks; four bottles were assigned to each inoculum (two with PEG and two without PEG). PEG was added on a substrate basis at a 1:1 ratio (*w*/*w*; PEG: sample) to ensure excess binding capacity relative to potential tannin content, as commonly applied in in vitro assays evaluating tannin biological activity.

At the end of incubation, total gas pressure and volume were measured using a digital pressure transducer (Delta Ohm DO9847, Selvazzano Dentro, Italy) and a calibrated syringe, following the methodology described by Theodorou et al. [36]. The gases were aspirated and stored in vacuum tubes until analysis. A 4.5 mL aliquot of fermentation fluid was collected from each bottle and acidified with 100 µL of sulfuric acid (H_2_SO_4_). Samples were used for volatile fatty acid analysis by gas–liquid chromatography and for ammonia concentration determination. An 800 µL aliquot of sample was mixed with 500 µL of a crotonic–metaphosphoric solution and injected into a Shimadzu GC 2010 (Kyoto, Japan), equipped with a flame ionization detector (FID) and a 30 m × 0.53 mm × 1 µm semi-capillary column (Supelco, Barcelona, Spain). Injector, detector, and oven temperatures were 240, 240, and 140 °C, respectively. Crotonic acid was used as an internal standard. Short-chain fatty acid (SCFA) production was calculated by subtracting the amount initially present in the inoculum from that measured at the end of the incubation period. Ammonia concentration was analyzed using colorimetry, following the technique of Weatherburn (1967) [37].

The incubation bottles were emptied, and the filter bags were removed, rinsed thoroughly under cold water, oven-dried at 60 °C, and weighed to determine in vitro DMD. Finally, the bags were subjected to neutral detergent extraction using an ANKOM fiber analyzer at 100 °C for 1 h according to Van Soest et al. [32] to determine the in vitro NDFD.

Data were analyzed using the PROC MIXED procedure of SAS (SAS software, Version 9.1; Copyright © 2002–2003 SAS Institute Inc., Cary, NC, USA) as a mixed model, including diet, treatment, and diet x treatment as fixed effects and inoculum as random effect. When a significant effect of treatment or diet (*p* < 0.05) was detected, differences between means were tested using Tukey’s multiple comparison test. Results were considered significant at *p* < 0.05.

## 3. Results

### 3.1. Chemical Composition Analysis

As expected, the chemical composition differed among the three diets. The S60 diet showed the highest NDF content (44.43%), followed by S40 (41.30%) and S20 (38.42%). A similar pattern was observed for ADF, with S60 displaying the highest value (20.56%) compared with S40 (18.97%) and S20 (19.05%). Conversely, S60 had the lowest ADL content (1.47%), whereas S20 exhibited the highest value (2.25%), likely due to the presence of alfalfa hay in the S20 diet. Furthermore, S20 had the highest CP content (13.98%), consistent with its inclusion of 20% alfalfa hay, well known for its high protein concentration. Finally, the three diets had comparable proportions of OM. The detailed chemical composition of the three tested diets is presented in Table 2.

### 3.2. In Vitro Digestibility

The in vitro digestibility results are shown in Table 3.

#### 3.2.1. Total Gas Production

Total gas production was not influenced by increasing sorghum inclusion levels in the diet or by the PEG treatment. Recorded values ranged from 123.25 to 133 mL, and no statistically significant differences were observed among treatments.

#### 3.2.2. Methane Production

Methane production was not affected by increasing sorghum inclusion levels (20%, 40%, and 60%), as no significant differences were detected among the experimental diets. Contrary to the conventional expectation that PEG-mediated neutralization of condensed tannins (CTs) would enhance methanogenesis, an overall significant reduction in methane production was observed between PEG-treated and untreated samples (*p* = 0.0158). However, Tukey’s post hoc test indicated that none of the pairwise comparisons (PEG vs. NO-PEG within each diet) showed a difference large enough to reach statistical significance.

#### 3.2.3. Dry Matter Digestibility (DMD)

As expected, DMD gradually declined with increasing proportions of sorghum in the diet, corresponding to higher levels of CTs. Accordingly, the diet containing 60% sorghum (S60) exhibited the lowest DMD (50.33%), whereas the S20 diet, (20% sorghum) recorded the highest value (54.55%), with the difference being highly significant (*p* = 0.0012). Furthermore, PEG supplementation did not significantly affect DMD (*p* > 0.05), although numerically higher DMD values were observed for the S60 diet.

#### 3.2.4. Neutral Detergent Fiber Digestibility (NDFD)

Surprisingly, NDFD increased significantly (*p* = 0.0005) with increasing sorghum inclusion. The three diets S20, S40, and S60 recorded values of 61.57%, 63.88%, and 70.15%, respectively. On the other hand, PEG treatment did not exert any significant effect on NDFD.

#### 3.2.5. Fermentation Products

1. Ammonia nitrogen (N-NH_3_)
  Consistent with expectations, PEG supplementation resulted in a statistically significant overall increase in N-NH_3_, as indicated by ANOVA (*p* = 0.042), reflecting a general difference between PEG-treated and untreated samples. However, Tukey’s post hoc test revealed that none of the individual diets exhibited significant pairwise differences. Moreover, no dose–response pattern was observed as the three experimental diets did not differ significantly from one another. Therefore, increasing the proportion of sorghum in the diet did not result in a reduction in N-NH_3_ concentration.
2. Volatile fatty acids (VFAs)
  Absolute concentrations and molar proportions of VFAs are presented in Table 4. Total VFA concentration was not significantly affected by increasing sorghum inclusion (*p* > 0.05). Likewise, PEG supplementation did not significantly influence total VFA concentration.

Furthermore, the molar proportion of isobutyrate (isoC4) decreased significantly (*p* < 0.0001) with increasing sorghum inclusion in the diet. This decrease was accompanied by a similar reduction in the absolute concentration of isobutyrate, indicating an overall decline in its production. Accordingly, the S60 diet exhibited the lowest values (1.35 without PEG and 1.75 with PEG). Additionally, PEG treatment significantly increased both the absolute concentration and the molar proportion of isobutyrate for diets S40 and S60 (*p* < 0.0001). However, no significant effect of PEG was observed for the S20 diet (*p* > 0.05).

In addition, increasing the sorghum inclusion rate significantly reduced both the absolute concentration (*p* = 0.0067) and the molar proportion (*p* = 0.0114) of butyric acid. The S20 and S40 diets showed comparable values, whereas the S60 diet recorded the lowest proportion (11.72%). In contrast, PEG treatment had no significant effect on butyrate molar proportion.

Although the absolute concentration of acetate was not significantly affected by sorghum inclusion level or PEG supplementation, its molar proportion was significantly altered. The molar proportion of acetate increased significantly (*p* = 0.0023) with increasing sorghum proportion in the diet. The S20 and S40 diets displayed similar values, whereas the S60 diet exhibited the highest proportion. In contrast, PEG addition had no significant effect on acetate molar proportion, as the variations observed between PEG-treated and untreated samples were minimal and not statistically significant.

Increasing sorghum inclusion in the diet resulted in a significant reduction in both the absolute concentration and the molar proportion of valerate (*p* = 0.0037) with the lowest values recorded for the S60 diet. Moreover, PEG addition led to an increase in valerate molar proportion; however, this effect was statistically significant only in the S20 and S40 diets (*p* = 0.0142).

Finally, the molar proportions of propionic acid, isovaleric acid, and caproic acid were not influenced by either increasing sorghum inclusion in the diet or by PEG treatment.

## 4. Discussion

Tannins have long been recognized as antinutritional compounds due to their adverse impact on voluntary feed intake, ruminal fiber degradation, and overall feed digestibility in ruminants [24,26]. Nevertheless, the effects of tannins can range from beneficial to detrimental, depending on their type, chemical structure, molecular weight, and the physiology of the consuming species [19,21,27,38,39]. In addition, PEG has a strong affinity for tannins, enabling it to bind these compounds and form stable tannin–PEG complexes, thereby neutralizing their biological activity. Consequently, PEG is widely used as an effective tool to highlight the effects specifically attributable to tannins present in forages [18,20,22,40]. Although the interaction between tannins and PEG has been extensively studied, limited information is available regarding the dose-dependent effects of increasing levels of sudangrass sorghum forage on rumen fermentation characteristics under feeding conditions representative of semi-arid Algerian regions. Within this framework, the present study aimed to evaluate the progressive incorporation of sudangrass sorghum (20%, 40%, and 60%) in ruminant diets and to assess its effects on in vitro digestibility, methane production, and volatile fatty acid profiles, both in the presence and absence of PEG.

Methane production

Dietary tannins have been widely recognized as a promising group of compounds for mitigating enteric methane emissions in ruminants [41,42]. Numerous in vivo and in vitro studies have reported that tannins naturally present in forages or supplied as tannin extracts can significantly reduce methane production [24,43,44,45]. Nevertheless, the literature reveals considerable variability in tannin effectiveness, particularly regarding their capacity to reduce ruminal methane formation per unit of digestible nutrient and the magnitude of the response observed [41].

Contrary to the initial hypothesis and to the methane-mitigating effects of tannins frequently reported in the literature, increasing sorghum inclusion levels (20%, 40%, and 60%) did not significantly affect methane production. No significant differences were observed among the experimental diets despite the expected concomitant increase in CTs content. These findings are in agreement with those of de Oliveira et al. [46], who reported that the inclusion of 40% sorghum in cattle diets did not alter methane production, regardless of the tannin content of the sorghum variety used. Similarly, Beauchemin et al. [47] observed that supplementing a forage-based diet with up to 1.8% CTs derived from quebracho extract (*Schinopis quebracho—colorado*) failed to suppress enteric methane emissions in growing cattle.

Given that acetate formation from pyruvate during ruminal fermentation is associated with hydrogen release, an increased acetate proportion generally tends to promote methanogenesis [41,48]. In the present study, the absence of methane-suppressing effect of sorghum tannins may partly be explained by the significant improvement in fiber digestibility (*p* = 0.0005) observed with increasing sorghum inclusion. This enhancement of fiber degradation was accompanied by a significant rise in acetate molar proportion (*p* = 0.0023). The resulting increase in hydrogen availability may have offset the potential inhibitory effect of tannins on methane formation.

On the other hand, in vitro studies have shown that tannins can exert antimethanogenic effects through both direct inhibition of methanogenic archaea and indirect mechanisms involving the suppression of ruminal protozoa [49]. Reduced protozoal activity is commonly associated with lower hydrogen transfer to methanogens and shifts in fermentation pathways, often reflected by changes in the VFA profiles, including a reduction in butyrate formation [50]. In the present study, this mechanism is supported by the significant decline in butyrate molar proportion (*p* = 0.0114) observed with increasing sorghum inclusion, suggesting an alteration of ruminal fermentation patterns associated with tannin presence. It is therefore plausible that these two contrasting effects on the hydrogen balance partially offset each other, leading to overall unchanged methane production, despite the presence of CTs.

Finally, the lack of more pronounced effects on methane production may also be attributed to the relatively low CTs content of the sorghum variety used in this study. This interpretation is consistent with the observations of Jayanegara et al. [41], who reported that low dietary levels of tannins are generally insufficient to consistently reduce methane emissions.

Furthermore, PEG supplementation, intended to neutralize tannins and thereby enhance methane production, actually showed a tendency to reduce methane output. Although the ANOVA indicated an overall significant difference (*p* = 0.0158), none of the pairwise comparisons reached statistical significance according to Tukey’s post hoc test. This suggests that the observed differences were modest and not consistently expressed across individual diets.

Our findings are in agreement with those of Mebirouk-Boudechiche et al. [51], who investigated the in vitro digestibility and fermentation kinetics of leaves from fodder shrubs collected in a pastoral area of northeastern Algeria and reported that PEG addition reduced methane production in *Phillyrea angustifolia* and *Phillyrea latifolia*. Comparable observations were reported by Singh et al. [52], who noted a decrease in gas production in *Leucaena leucocephala* and *Melia azedarach* following PEG addition after 24 h of incubation. In the same context, Sisay et al. [53] also reported no significant effect of PEG on gas and methane production in *Prosopis juliflora, Vernonia amygdalina*, and *Croton macrostachyus*.

The limited effect of PEG on methane production observed in our experiment may be attributed to the relatively low CTs content of the sorghum variety used, which likely limited the extent to which PEG could effectively neutralize tannin activity.

Dry Matter Digestibility

In this study, DMD declined progressively as sorghum inclusion increased, reflecting the concurrent rise in CTs levels. The S60 diet exhibited the lowest DMD (50.33%), whereas the S20 diet recorded the highest value (54.55%), with this difference being highly significant (*p* = 0.0012).

These results are consistent with the meta-analysis conducted by Jayanegara and Palupi [54], which demonstrated a linear decrease in OM digestibility, both in vitro and in vivo, with increasing dietary CTs levels. Similarly, Teixeira et al. [55] reported a reduction in DMD in sheep fed sorghum silage associated with higher CTs content. Comparable trends have also been observed in vitro, as Zhang et al. [56] documented a significant reduction in DMD with increasing proportions of sweet sorghum in silage mixtures. More recently, the meta-analysis by Al Rharad et al. [57] further confirmed that dietary tannins negatively affect multiple digestibility parameters, including DMD in small ruminants. However, Carneiro et al. [58] reported that silages derived from different sorghum genotypes with CTs concentrations below 10.62% did not exert a depressive effect on in vitro DMD.

Overall, these observations support the idea that tannins reduce feed digestibility through the formation of complexes with natural polymers such as proteins and polysaccharides, thereby limiting their accessibility to rumen microorganisms [19,20,21,22,24,59]. Notably, evidence suggests that the reduction in in vitro OM digestibility may be particularly pronounced at relatively low concentrations of CTs [54].

With respect to PEG treatment, no significant effect on DMD was observed in the present study. Similar results to ours were reported by Kondo et al. [60], who observed that PEG supplementation did not affect the in vitro OM digestibility of tannin-rich green tea by-products. Likewise, Sisay et al. [53] reported no effect on OM digestibility in forages characterized by low CTs content. In the same context, Xie et al. [18] found that PEG inclusion in diets based on tannin-rich grain sorghum had no significant impact on either DM or OM digestibility in cattle.

These findings contrast with several reports in the literature indicating improvements in dry matter and OM digestibility following tannin neutralization with PEG. For instance, Kamalak et al. [40] reported that OM digestibility of tannin-containing tree leaves increased proportionally with PEG supplementation. Similarly, Gemeda et al. [43] reported a significant enhancement of in vitro OM digestibility following PEG addition to a range of tannin-rich forage plants. Likewise, Kondo et al. [60] demonstrated a marked increase in in vitro OM digestibility following PEG supplementation to tannin-rich black tea by-products.

This variability among studies suggests that the diversity of the chemical composition of tannins present in forages strongly influences their reactivity to PEG [40]. Such variability may help explain the absence of a significant PEG effect on DMD observed in the present experiment.

Neutral detergent fiber digestibility

Interestingly, NDFD increased significantly (*p* = 0.0005) with higher sorghum inclusion in the diet. The S20, S40, and S60 diets exhibited NDFD values of 61.57%, 63.88%, and 70.15%, respectively.

Our results contrast with those reported by de Oliveira et al. [46], who observed that increasing tannin content in forage sorghum included at 40% of the diet negatively affected ruminal NDF degradation. Similarly, Cabral Filho et al. [61] reported a significant decline in NDFD in sheep fed a high-tannin grain sorghum cultivar compared with low-tannin cultivars. Consistent with these observations, Teixeira et al. [55] reported a reduction in NDFD in sheep as tannin levels increased across different sorghum silage varieties.

In our study, the observed increase in NDFD appears to be primarily related to differences in diet composition. Specifically, the S20 diet contained a higher lignin concentration (2.25%) than the S40 (1.50%) and S60 (1.47%) diets, likely due to the combined inclusion of alfalfa hay (20%) and corn silage (20%). Because lignin is a major limiting factor in plant cell wall degradation [62], the progressive diminution in its level from S20 to S60 could explain the increase in NDFD despite the concurrent rise in CTs levels.

Moreover, in the present experiment, adding PEG did not affect NDFD. Similar findings were reported by Abarghuei et al. [63], who stated that neutralization of oak leaf tannins through PEG supplementation had no measurable influence on NDFD. In contrast, Xie et al. [18] observed a tendency toward increased NDFD when PEG was added to diets based on tannin-rich grain sorghum in cattle.

The absence of a significant PEG effect on NDFD suggests that, under the conditions of the present study, the potential inhibition of fibrolysis by tannins was limited. This observation may be explained by the fact that CTs generally exert less pronounced antinutritional effects on fiber digestibility than on protein digestibility [64].

Furthermore, Mueller-Harvey [65] emphasized that when tannins are incorporated into mixed diets, their antinutritional effects are often attenuated compared with those observed in highly tannin-rich forages. This attenuation may also explain the absence of a more pronounced response to PEG in the present study. Moreover, tannins display substantial structural heterogeneity across plant species, and even slight modifications in their molecular structures can markedly alter their biological effects [65].

Protein degradability

The effects of tannins on ruminal nitrogen metabolism are well documented. Tannins bind to dietary proteins in the rumen, causing their precipitation and thus limiting their excessive degradation by microorganisms [19,24,27,66]. This reduction in protein degradation results in a decrease in N-NH_3_ production [24,63,67].

However, under the conditions of the present study, the N-NH_3_ results did not support this hypothesis. Increasing the proportion of sorghum in the diet, and consequently raising CTs levels, did not result in a significant variation N-NH_3_. No significant differences were detected among the three experimental diets, indicating the absence of a dose–response relationship.

Similar observations were reported by de Oliveira et al. [46], who demonstrated that incorporating sorghum silage at 40% of the diet did not alter N-NH_3_ concentration in cattle, irrespective of the tannin content of the sorghum variety used. Likewise, Tian et al. [68] found no significant difference in NH_3_ concentrations in rumen fluid during in vitro fermentation of stems from six sorghum varieties differing in their phenolic compound profiles.

According to Apajalahti et al. [69], although N-NH_3_ is commonly used as a marker of protein degradation in the rumen, it is not a final product of nitrogen metabolism, and its residual concentration alone is not a sufficiently sensitive indicator of the extent of protein degradation. In this regard, monitoring the molar proportions of branched-chain volatile fatty acids, which originate from the deamination of branched-chain amino acids, provides a more sensitive and reliable approach for assessing microbial proteolytic activity in the rumen [69].

Branched-chain volatile fatty acids (isoSCFAs), mainly isobutyrate and isovalerate, arise from the deamination of branched-chain amino acids and, similarly to N-NH_3_, are widely recognized as markers of the extent of protein degradation in the rumen [54,67,70,71]. Numerous in vitro trials have shown that increasing dietary levels CTs are generally associated with a significant reduction in ruminal ammonia (NH_3_) and isoSCFA production, although this response is often nonlinear [54].

In this experiment, the molar proportion of isobutyrate decreased considerably (*p* < 0.0001) with increasing sorghum inclusion, reflecting the concomitant rise in CTs levels. This response suggests an effective reduction in valine deamination and, consequently, a lower extent of ruminal protein degradation under the influence of tannins. Isobutyrate is considered a more sensitive indicator of branched-chain amino acid deamination than N-NH_3_ concentration [69].

In the current study, the capacity of sorghum tannins to modulate protein degradation was further supported by the overall significant increase N-NH_3_ observed following PEG supplementation (*p* = 0.042). This response suggests effective neutralization of sorghum tannins by PEG preventing the formation of protein–CT complexes and consequently enhancing protein availability to proteolytic bacteria in the rumen.

Our findings are consistent with those of Xie et al. [18], who noted a slight increase in ruminal N-NH_3_ concentration and the molar proportion of isovalerate following PEG supplementation in steers fed tannin-rich sorghum grain. Similarly, Kondo et al. [60] showed that the addition of PEG to green and black tea by-products significantly increased the N-NH_3_ concentration in the fermentation medium.

Concurrently, PEG treatment significantly increased (*p* < 0.0001) the molar proportion of isobutyrate in the S40 and S60 diets, indicating enhanced deamination of branched-chain amino acids and, consequently, a higher intensity of proteolysis when tannins were neutralized. However, no PEG effect on isobutyrate molar proportion was observed for the S20 diet. This lack of response may be explained by its chemical composition, characterized by a lower tannin content and greater baseline protein availability, likely associated with the inclusion of corn silage and, more specifically, alfalfa hay.

It should be emphasized that, because CTs were not analytically quantified in this study, the interpretation of our results relies on their biological activity as revealed by PEG supplementation. The consistent increase in N-NH_3_ and isobutyric acid molar proportion following PEG addition provides functional evidence of moderate yet biologically relevant tannin–protein interactions.

Volatile Fatty Acid production

Although the absolute concentration of total VFA was not significantly affected by the increasing sorghum inclusion or by PEG supplementation, marked changes were observed in the molar proportions of individual VFA. This pattern indicates a qualitative shift in fermentation pathways rather than an overall reduction in the intensity of ruminal fermentation. This stability of total VFA production suggests that the level of CTs present in the sorghum variety used in this experiment was insufficient to decrease fermentation activity but sufficient to modulate microbial metabolism. This observation is consistent with several in vitro studies reporting that moderate tannin levels alter fermentation profiles without significantly affecting total VFA production [64,72].

Furthermore, the significant increase in acetate molar proportion observed with higher sorghum inclusion is likely related to the simultaneous improvement in fiber digestibility. Indeed, enhanced degradation of plant cell walls generally promotes acetate production, as cellulolytic bacteria mainly direct structural carbohydrate fermentation toward this pathway [73]. This response suggests that changes in diet composition, particularly the progressive decrease in lignin content from S20 to S60, may have contributed to the observed shifts in acetate proportion, irrespective of the presence of CTs.

Conversely, the significant decrease in butyrate molar proportion with the increasing sorghum inclusion is consistent with the findings of the meta-analysis by Jayanegara et al. [64], which reported reduced butyrate proportions in several studies assessing the effect of tannins on in vitro ruminal fermentations. This decrease suggests selective inhibition of butyrogenic pathways, given that butyrate formation is closely associated with protozoal activity [50] and protozoa are known to be particularly sensitive to tannins [49].

Moreover, branched-chain VFA, particularly isobutyrate, exhibited marked responses to both increasing sorghum incorporation and PEG supplementation, in agreement with the meta-analysis by Jayanegara et al. [41]. Since isobutyrate originates from the deamination of valine, its decrease with increasing sorghum levels reflects a reduction in ruminal proteolysis, probably related to the formation of tannin–protein complexes [54,69].

The significant increase in isobutyrate concentration and molar proportion following PEG addition further confirms the biological activity of CTs and highlights their role in modulating ruminal nitrogen metabolism.

In addition, the absolute concentrations of certain individual VFA, particularly butyric acid, isobutyric acid, and valeric acid, were significantly affected by diet composition and/or PEG treatment.

The concomitant variations observed in both the molar proportions of VFA and the absolute concentrations of certain individual VFA, suggest a reorientation of microbial metabolic pathways induced by CTs present in sorghum, rather than a generalized inhibition of overall fermentation activity.

## 5. Conclusions

This study demonstrates that increasing the proportion of sudangrass sorghum forage in ruminant diets modifies in vitro rumen fermentation characteristics, mainly due to the effects of CTs. Higher levels of sorghum led to reduced dry matter digestibility, while NDFD increased. This increase appears to be associated with differences in lignin concentration among diets rather than a direct tannin effect.

Despite the increase in CTs content, methane production was not significantly impacted by higher sorghum proportions. Changes in the volatile fatty acid profiles, particularly increased acetate and reduced butyrate proportions, suggest alterations in fermentation pathways that may offset the potential antimethanogenic effect of tannins.

The addition of PEG resulted in higher N-NH_3_ concentrations and increased isoSCFA levels, supports the involvement of tannin–protein interactions and their influence on rumen protein metabolism.

Overall, these findings suggest that the CTs in the sorghum forage used in this study have moderate, yet biologically relevant, effects on rumen fermentation without severely impairing overall fermentative activity. Thus, sudangrass sorghum can be considered a suitable forage resource for ruminant feeding systems in semi-arid regions, though its nutritional impact depends on the level of inclusion and the composition of the diet. Further studies combining in vivo approaches and tannin characterization would elucidate the long-term implications of sorghum tannins on animal performance and methane mitigation.

## Figures and Tables

**Table 1:** Composition of experimental diets (% DM).

Diet	Sorghum (S)	Corn Silage (CS)	Alfalfa Hay (L)	Concentrate (C)
S20	20	20	20	40
S40	40	20	-	40
S60	60	-	-	40

**Table 2:** Chemical composition (g/kg DM) of experimental diets.

Diet	NDF	ADF	ADL	OM	CP
S20	384.2	190.5	22.5	940.5	139.8
S40	413.0	189.7	15.0	946.9	122.1
S60	444.3	205.6	14.7	945.7	121.0

NDF: neutral detergent fiber; ADF: acid detergent fiber; ADL: acid detergent lignin; CP: crude protein; OM: organic matter.

**Table 3:** Gas production, methane production, dry matter digestibility, neutral detergent fiber digestibility, and ammonia nitrogen concentration after 24 h of in vitro fermentation of experimental diets with or without polyethylene glycol (PEG).

Variable	Treatment	S20	S40	S60	SEM	P-Diet	P- Treatment	P-Diet × Treatment
Gas (mL)	PEG	125	123	123	9.28	0.9851	0.3185	0.9373
	NO	128	133	132				
Methane (mmol)	PEG	5.04	4.94	4.89	0.27	0.7792	0.0158	0.3613
	NO	5.13	5.49	5.44				
DMD (%)	PEG	54.8	51.9	52.7	1.16	0.0012	0.1294	0.2826
	NO	54.5 ^a^	51.7 ^ab^	50.3 ^b^				
NDFD (%)	PEG	61.9 ^b^	64.4 ^ab^	71.2 ^a^	1.87	0.0005	0.6242	0.9813
	NO	61.5 ^b^	63.8 ^ab^	70.1 ^a^				
N-NH_3_ (mg/L)	PEG	510	495	471	52.08	0.5378	0.042	0.5119
	NO	464	440	461				

DMD: dry matter digestibility; NDFD: neutral detergent fiber digestibility; N-NH_3_: ammonia nitrogen; SEM: standard error of the mean. Means within a row with different superscripts (a, b) differ (*p* < 0.05; Tukey’s test).

**Table 4:** Production and molar proportions of volatile fatty acids (VFAs) after 24 h of in vitro fermentation of experimental diets with or without polyethylene glycol (PEG).

Variable	Treatment	S20	S40	S60	SEM	P-Diet	P- Treatment	P-Diet × Treatment
VFA (µmol)	PEG	5260	5059	5175	276.5	0.1110	0.3654	0.3493
	NO	5512	5217	5058				
Ac (µmol)	PEG	3116	3051	3219	80.3	0.2852	0.3555	0.1566
	NO	3333	3133	3107				
Prop (µmol)	PEG	1059	992	972	23.1	0.0557	0.1647	0.7483
	NO	1067	1034	1014				
Isob (µmol)	PEG	110 ^a^	94.2 ^b^*	90.2 ^b^*	3.24	<0.0001	<0.0001	0.0922
	NO	104 ^a^	75.5 ^b^*	68.2 ^b^*				
But (µmol)	PEG	650	627	600	20.8	0.0067	0.0765	0.3378
	NO	721 ^a^	652 ^ab^	605 ^b^				
Isoval (µmol)	PEG	187	143	195	7.3	0.1807	0.9711	0.0253
	NO	199	187	168				
Val (µmol)	PEG	102	92.5	92.2	2.78	0.0037	0.3109	0.8213
	NO	100	91.2	87.7				
Cap (µmol)	PEG	2.75	9.50	6.00	1.98	0.4315	0.4694	0.3859
	NO	8.00	7.75	7.00				
Others (µmol)	PEG	403	303	384	63.8	0.3857	0.5438	0.1535
	NO	350	361	331				
Ac/Pr	PEG	2.77	3.05	3.30	0.15	0.0792	0.37	0.1014
	NO	2.92	2.81	3.06				
(mol/100 mol)								
P-ac	PEG	59.2 ^b^	60.2 ^b^	62.2 ^a^	0.70	0.0023	0.8095	0.4883
	NO	59.9 ^b^	60.0 ^b^	61.5 ^a^				
P-prop	PEG	21.5	20.0	19.0	0.80	0.2144	0.4013	0.0465
	NO	20.6	21.5	20.2				
P-isob	PEG	2.12 ^a^	1.87 ^ab^*	1.75 ^b^*	0.11	<0.0001	<0.0001	0.2089
	NO	1.91 ^a^	1.45 ^b^*	1.35 ^b^*				
P-but	PEG	12.1 ^a^	12.1 ^a^	11.3 ^b^	0.78	0.0114	0.0911	0.5531
	NO	12.9 ^a^	12.3 ^a^	11.7 ^b^				
P-isoval	PEG	3.53	3.22	3.70	0.27	0.9846	0.1597	0.0756
	NO	3.25	3.52	3.28				
P-val	PEG	1.96 ^a^*	1.83 ^ab^*	1.79 ^b^	0.12	0.0037	0.0142	0.4449
	NO	1.86 ^a^*	1.77 ^b^*	1.75 ^b^				
P-cap	PEG	0.06	0.18	0.10	0.07	0.4489	0.5995	0.5982
	NO	0.12	0.14	0.13				
P-others	PEG	7.68	6.29	7.35	0.36	0.2737	0.5258	0.3054
	NO	7.15	6.90	6.51				

VFA: volatile fatty acid; Ac: acetate; Prop: propionate; Isob: isobutyrate; But: butyrate; Isoval: isovalerate; Val: valerate; Cap: caproate; others: minor volatile fatty acids not individually identified or quantified; P-: molar proportion; SEM: standard error of the mean. Means within a row with different superscripts (a, b) differ (*p* < 0.05; Tukey’s test). * indicates differences between PEG and NO PEG treatments (*p* < 0.05; Tukey’s test).

## Data Availability

Data are contained within the article.
